# Virus-host immune interaction in asymptomatic HTLV-1 carriers

**DOI:** 10.1128/spectrum.02507-24

**Published:** 2025-01-31

**Authors:** Theodore Worlanyo Asigbee, Midori Nakamura-Hoshi, Nozomi Kuse, Hiroshi Ishii, Koichi Ishikawa, Ai Kawana-Tachikawa, Erika Horibe, Makoto Nakashima, Yoshihisa Yamano, Kaoru Uchimaru, Tetsuro Matano

**Affiliations:** 1 Graduate School of Medical Sciences and Joint Research Center for Human Retrovirus Infection, Kumamoto University, Kumamoto, Japan; 2 AIDS Research Center, National Institute of Infectious Diseases, Tokyo, Japan; 3 Research Center for Drug and Vaccine Development, National Institute of Infectious Diseases, Tokyo, Japan; 4 Department of Rare Diseases Research, Institute of Medical Science, St. Marianna University School of Medicine, Kawasaki, Japan; 5 Department of Computational Biology and Medical Sciences, Graduate School of Frontier Sciences, University of Tokyo, Tokyo, Japan; 6 Institute of Medical Science, University of Tokyo, Tokyo, Japan; University of Miami, Miami, Florida, USA

**Keywords:** HTLV-1, antibody response, neutralization activity, T-cell immunity

## Abstract

Human T-cell leukemia virus type 1 (HTLV-1) induces chronic long-term latent infection that can cause fatal diseases, including adult T-cell leukemia. HTLV-1 production is poor and undetectable during the asymptomatic phase of infection. Virus-host immune interaction in latent infection has not been fully determined. In the present study, virus-specific antibody and T-cell responses in asymptomatic HTLV-1 carriers (*n* = 77) were investigated. Neutralizing antibody responses were significantly higher in individuals with higher anti-Env SU (gp46) antibody levels and positively correlated with proviral loads. Overnight *ex vivo* culture of peripheral blood mononuclear cells resulted in detectable HTLV-1 Gag antigen (p19) expression in CD4^+^ T cells and interferon-γ (IFN-γ) induction in CD8^+^ T cells. Frequencies of these p19-expressing CD4^+^ T cells and non-specific IFN-γ-induced CD8^+^ T cells were positively correlated with proviral loads, respectively. Tax-specific CD8^+^ T-cell frequencies were not associated with proviral loads but inversely correlated with the ratios of p19-expressing CD4^+^ T-cell frequencies to proviral loads, implying the involvement of Tax-specific CD8^+^ T cells in the control of HTLV-1 replication. These results provide insights into the elucidation of the virus-host immune interaction in latent HTLV-1 infection.

**IMPORTANCE:**

Human T-cell leukemia virus type 1 (HTLV-1) can cause fatal diseases, including adult T-cell leukemia in humans after long-term asymptomatic infection. In asymptomatic HTLV-1 carriers, substantial proviruses are detectable in lymphocytes, and the association of a higher proviral load with a higher risk of disease progression has been observed. However, viral replication is controlled and HTLV-1 production is poor in asymptomatic carriers. Virus-host immune interaction during latent infection has not been fully determined. In the present study, virus-specific antibody and T-cell responses in asymptomatic HTLV-1 carriers were investigated. Neutralizing antibody responses were positively correlated with proviral loads. Tax-specific CD8^+^ T-cell frequencies were not associated with proviral loads but inversely correlated with the ratios of p19-expressing CD4^+^ T-cell frequencies to proviral loads, supporting the notion that Tax-specific CD8^+^ T-cell responses play an important role in the control of HTLV-1 replication. These results provide insights into the mechanism of virus-host immune interaction in latent HTLV-1 infection.

## INTRODUCTION

Human T-cell leukemia virus type 1 (HTLV-1) induces chronic asymptomatic infection in humans (1, 2). An estimated 5–10 million people globally are infected with HTLV-1, although the actual number is probably higher due to a lack of reliable epidemiologic data (3). A small number of HTLV-1-infected carriers develop severe diseases, including adult T-cell leukemia (ATL) and HTLV-1-associated myelopathy or tropical spastic paraparesis after long-term asymptomatic infection (4–6). The exact mechanism for developing diseases in HTLV-1 carriers has not been fully elucidated (7).

HTLV-1 infection is latent in asymptomatic carriers, and viral structural protein expression and viral replication were poor *in vivo* (8–10). Expression of viral Tax and structural proteins is mostly undetectable in freshly prepared peripheral blood mononuclear cells (PBMCs) derived from HTLV-1 carriers but becomes detectable after *ex vivo* cell culture (11–15). Therefore, the host is considered to have a mechanism to control HTLV-1 replication *in vivo*.

Proviruses are detectable in PBMCs of asymptomatic HTLV-1 carriers. Proviral load (PVL) varies among carriers but serves as a predictive marker for the prognosis of associated diseases, and carriers with high PVL of ≥4 copies per 100 PBMCs are at a higher risk of developing ATL (16). Thus, elucidation of the viral control mechanism in asymptomatic carriers is an important issue.

Upon HTLV-1 transmission, latently infected cells are transmitted from infected to uninfected individuals. This results in viral expression and cell-to-cell HTLV-1 transmission from the donor’s infected cells to recipient cells, followed by further cell-to-cell transmission from the newly infected recipient cells to uninfected cells, leading to the establishment of HTLV-1 infection. It is speculated that viral expression occurs and induces host immune responses during this acute phase of infection. These host immune responses may possibly contribute to the control of HTLV-1 replication, resulting in latent HTLV-1 infection. Understanding this virus-host immune interaction is important for elucidating the viral control mechanism in asymptomatic HTLV-1 carriers.

Previous studies have indicated the involvement of CD8^+^ cells, particularly virus-specific CD8^+^ T cells, in the control of HTLV-1 replication (17–20). In particular, HTLV-1 Tax antigen-specific CD8^+^ T-cell responses have been implicated in HTLV-1 control (21–25). We have recently shown that CD8^+^ cell depletion by monoclonal anti-CD8 antibody administration results in HTLV-1 expression and an increase in proviral load in HTLV-1-infected cynomolgus macaques (26). Our results indicate that HTLV-1 can proliferate (via HTLV-1 cell-to-cell transmission and/or replication of HTLV-1-infected cells) in the absence of CD8^+^ cells, suggesting that CD8^+^ cells are responsible for the control of HTLV-1 replication. This implies that virus-host immune interaction occurs in the chronic phase of HTLV-1 infection.

In the present study, virological and immunological analyses were performed using PBMCs obtained from HTLV-1-infected carriers. PVL, viral antigen expression in *ex vivo* culture, antibody responses, and T-cell responses were investigated. PVL was not associated with Tax-specific CD8^+^ T-cell frequencies. However, the ratios of viral antigen-expressing CD4^+^ T-cell frequencies to PVL were inversely correlated with Tax-specific CD8^+^ T-cell frequencies, confirming the involvement of Tax-specific CD8^+^ T cells in the control of HTLV-1 replication. Accumulation of these data would contribute to our understanding of the virus-host immune interaction in latent HTLV-1 infection.

## RESULTS

### Asymptomatic HTLV-1-infected carriers

**Fig 1:**
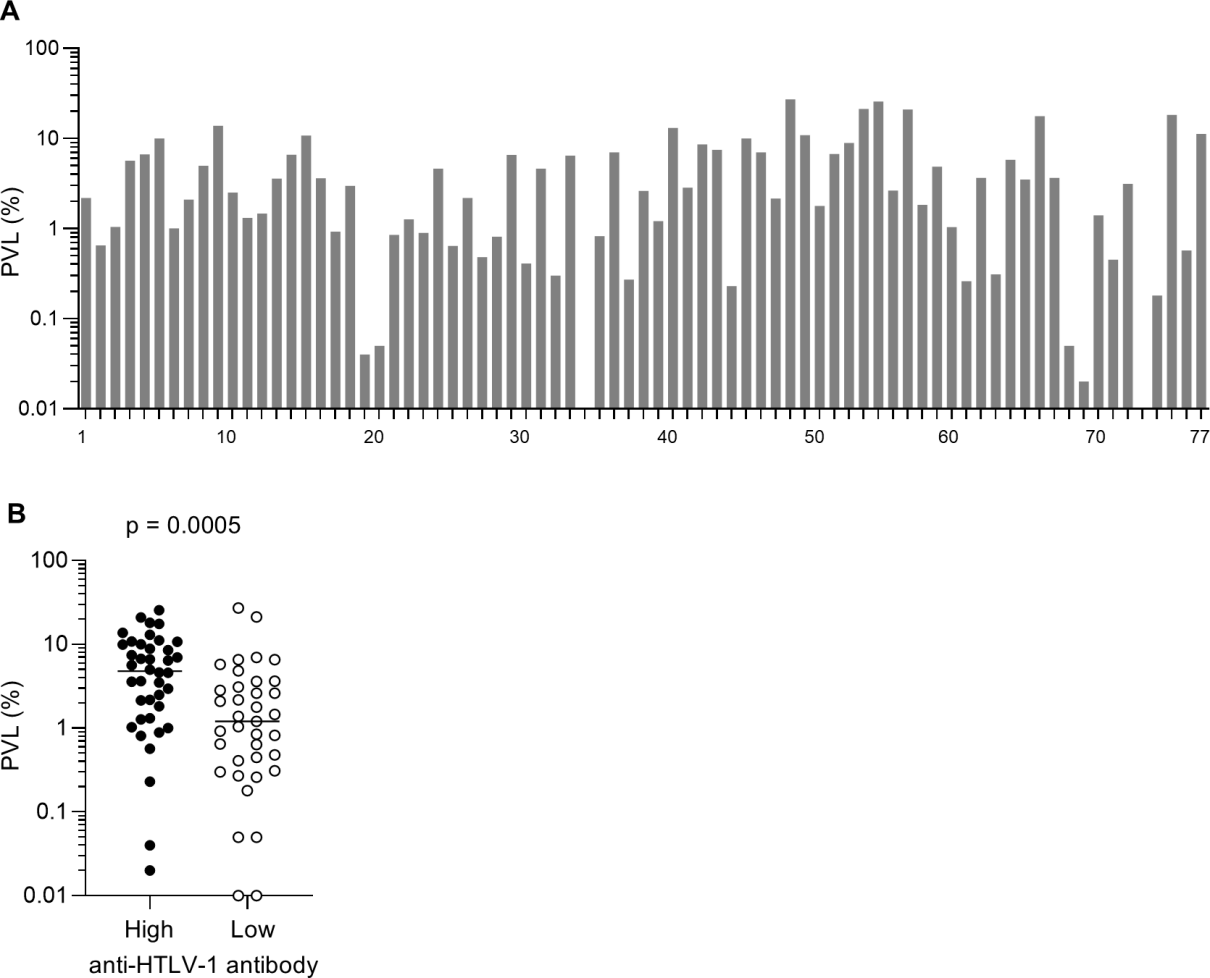
Proviral loads in asymptomatic HTLV-1 carriers. (**A**) PVLs in asymptomatic HTLV-1 carriers (*n* = 77). (**B**) Comparison of PVLs between carriers with high and low anti-HTLV-1 antibody levels.

A total of 77 asymptomatic HTLV-1-infected carriers positive for anti-HTLV-1 antibodies were enrolled in the present study. The median age of all participants was 59 years (IQR: 49–65), and 51 (66%) were females. The geometric mean of their PVLs was 0.62% (Fig. 1A). PVLs and anti-HTLV-1 antibodies were determined in all 77 participants, whereas assessment of HTLV-1 p19 expression, interferon-γ (IFN-γ) induction, and Tax-specific CD8^+^ T-cell responses was limited to 53 individuals because of the limitation of sample availability.

### Anti-HTLV-1 antibody responses

Plasma anti-Gag (p19 and p24) and anti-Env (gp46 and gp21) antibodies were assessed by line immunoassay. Based on the band scores reflecting levels of antibodies detected by line immunoassay, participants were divided into two groups: individuals showing band scores greater than or equal to 3+ in either anti-p19, anti-p24, anti-gp46, or anti-gp21 were classified as those with high anti-HTLV-1 antibody responses, whereas the remaining were categorized as those with low antibody responses. Comparison of PVLs between carriers with high and low anti-HTLV-1 antibody levels revealed significantly higher PVL in the former (*P* = 0.0005 by Mann-Whitney *U* test) (Fig. 1B).

**Fig 2:**
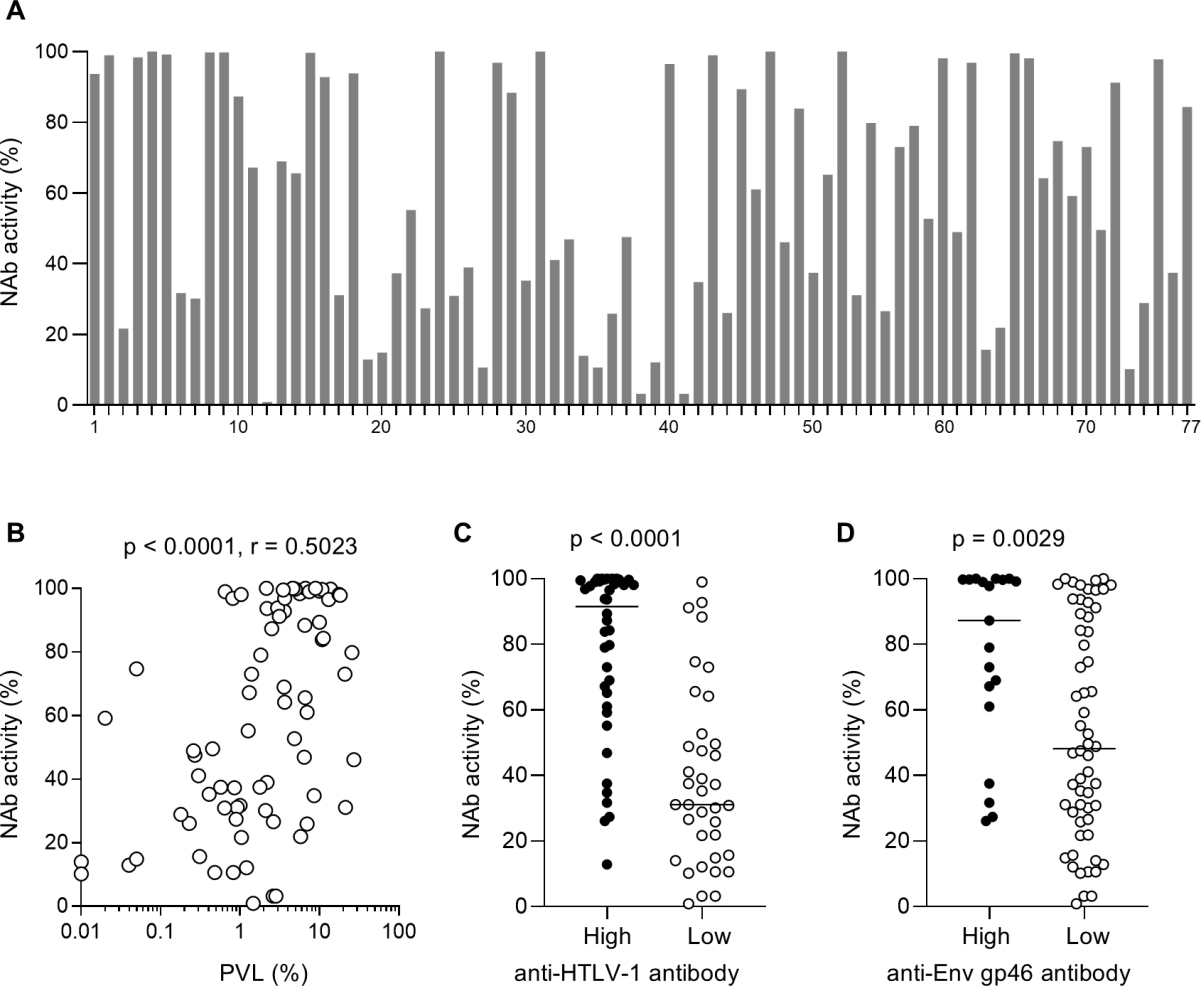
Neutralizing antibody activity in asymptomatic HTLV-1 carriers. (**A**) Plasma NAb activity in asymptomatic HTLV-1 carriers (*n* = 77). Syncytia inhibition activities of 200-fold diluted plasma samples are shown. (**B**) Correlation between PVL and NAb activity. (**C**) Comparison of NAb activity between carriers with high and low anti-HTLV-1 antibody levels. (**D**) Comparison of NAb activity between carriers with high and low anti-HTLV-1 gp46 antibody levels.

Anti-HTLV-1 neutralizing antibody (NAb) activity was examined by syncytia inhibition assay (Fig. 2A). Most of the HTLV-1 carriers with NAb inhibitory activity higher than 50% in 200-fold diluted plasma had PVL higher than 1%. A significant positive correlation between PVL and NAb activity was shown (*P* < 0.0001, *r* = 0.5023 by Spearman’s test) (Fig. 2B). Individuals with high anti-HTLV-1 antibody levels exhibited significantly higher NAb activity than those with low anti-HTLV-1 antibody levels (*P* < 0.0001 by Mann-Whitney *U* test) (Fig. 2C). Also, individuals with high anti-HTLV-1 Env SU (gp46) antibody levels exhibited significantly higher NAb activity (*P* = 0.0029 by Mann-Whitney *U* test) (Fig. 2D). Thus, antibody responses were associated with PVLs.

### HTLV-1 antigen expression in *ex vivo* culture

**Fig 3:**
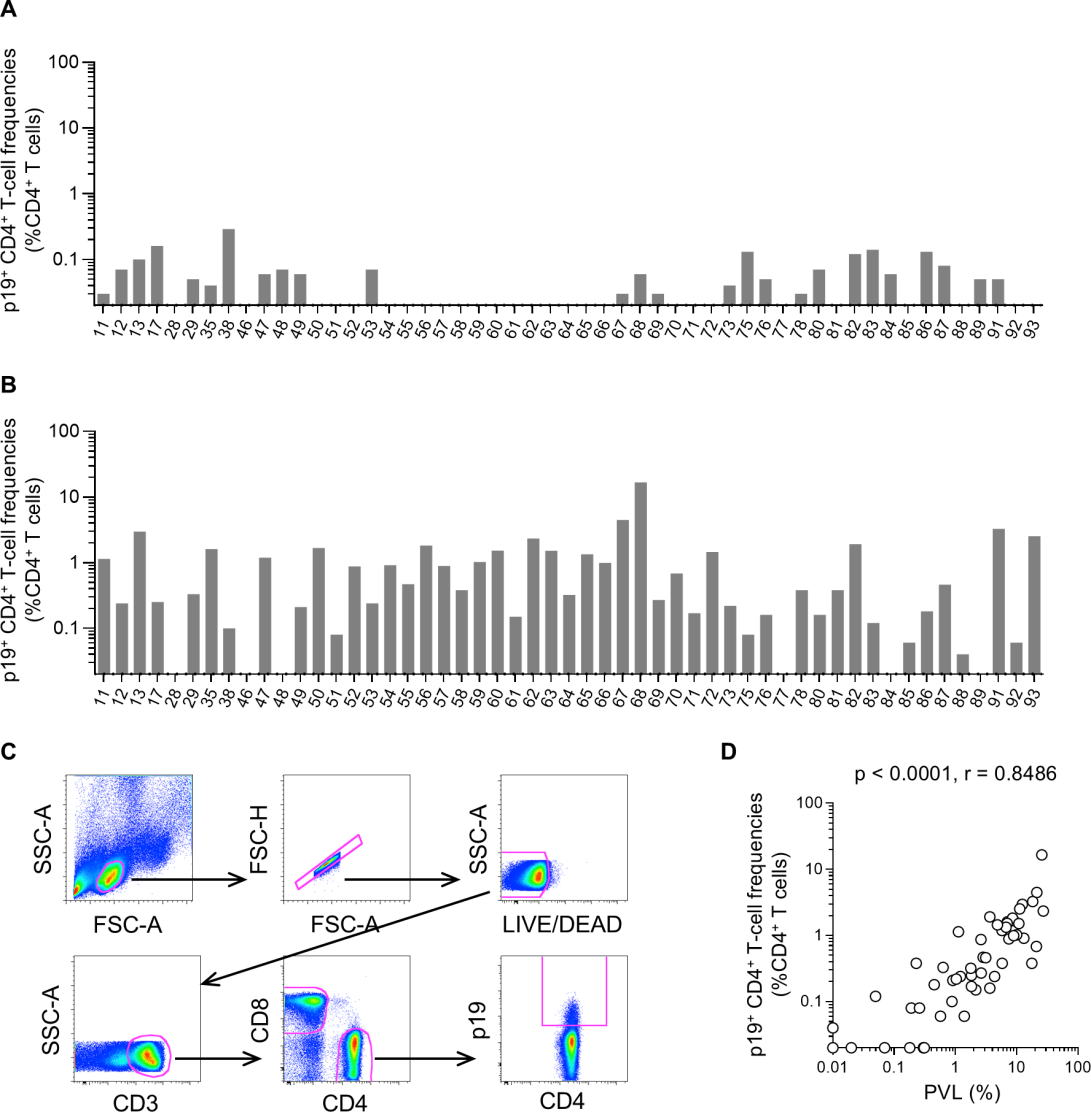
Frequencies of p19-expressing CD4^+^ T cells in asymptomatic HTLV-1 carriers. (**A**) Frequencies of p19-expressing CD4^+^ T cells freshly prepared from asymptomatic HTLV-1 carriers (*n* = 53). (**B**) Frequencies of CD4^+^ T cells expressing p19 after overnight culture of PBMCs obtained from carriers. (**C**) A representative gating schema for the detection of p19 expression after overnight culture in flow cytometric analysis. Data on PBMCs of sample #91 are shown. (**D**) Correlation between PVLs and p19-expressing CD4^+^ T-cell frequencies.

In the HTLV-1 replication process, Tax expression is the earliest, triggering the expression of structural proteins. It is known that viral structural protein expression is mostly undetectable in freshly prepared PBMCs derived from HTLV-1 carriers but becomes detectable after *ex vivo* cell culture (11, 13). Thus, using PBMCs derived from asymptomatic HTLV-1 carriers (*n* = 53), we examined HTLV-1 Gag matrix protein (p19) expression both in freshly prepared PBMCs and PBMCs after overnight culture. The p19 is a Gag protein that is sensitive to detection and has been used as an indicator of HTLV-1 production (25). Flow cytometric analysis following intracellular immunostaining was performed. Expression of p19 was poor in freshly prepared PBMCs without *ex vivo* culture but became detectable in a majority of carriers (48/53) after overnight culture (Fig. 3A through C). A significant positive correlation was observed between PVLs and frequencies of p19-expressing CD4^+^ T cells after overnight cell culture (*P* < 0.0001, *r* = 0.8486 by Spearman’s test) (Fig. 3D).

**Fig 4:**
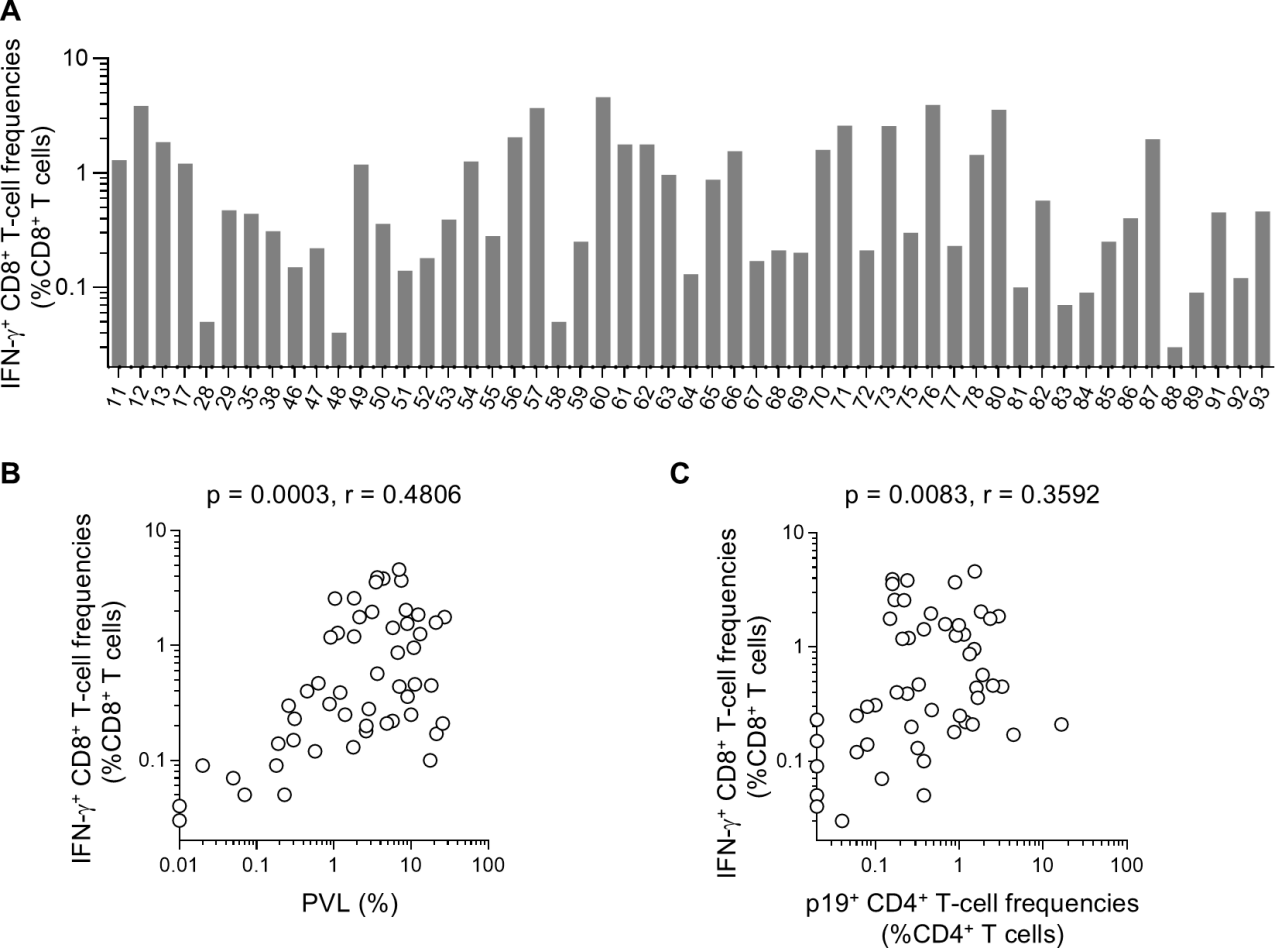
IFN-γ induction in CD8^+^ T cells obtained from asymptomatic HTLV-1 carriers. (**A**) Frequencies of IFN-γ^+^ CD8^+^ T cells after overnight culture of PBMCs obtained from asymptomatic HTLV-1 carriers (*n* = 53). (**B**) Correlation between PVLs and IFN-γ^+^ CD8^+^ T-cell frequencies. (**C**) Correlation between p19-expressing CD4^+^ T-cell frequencies and IFN-γ^+^ CD8^+^ T-cell frequencies.

We then examined non-specific IFN-γ induction (without specific stimulation) in CD8^+^ T cells after overnight culture. Non-specific IFN-γ^+^ CD8^+^ T-cell induction was detected in all samples after overnight culture (Fig. 4A). A significant positive correlation was observed between PVLs and the IFN-γ^+^ CD8^+^ T-cell frequencies (*P* = 0.0003, *r* = 0.4806 by Spearman’s test) (Fig. 4B). A significant positive correlation was also observed between frequencies of p19-expressing CD4^+^ T cells and IFN-γ^+^ CD8^+^ T cells after overnight cell culture (*P* = 0.0083, *r* = 0.3592 by Spearman’s test) (Fig. 4C).

### Tax-specific CD8^+^ T-cell responses

**Fig 5:**
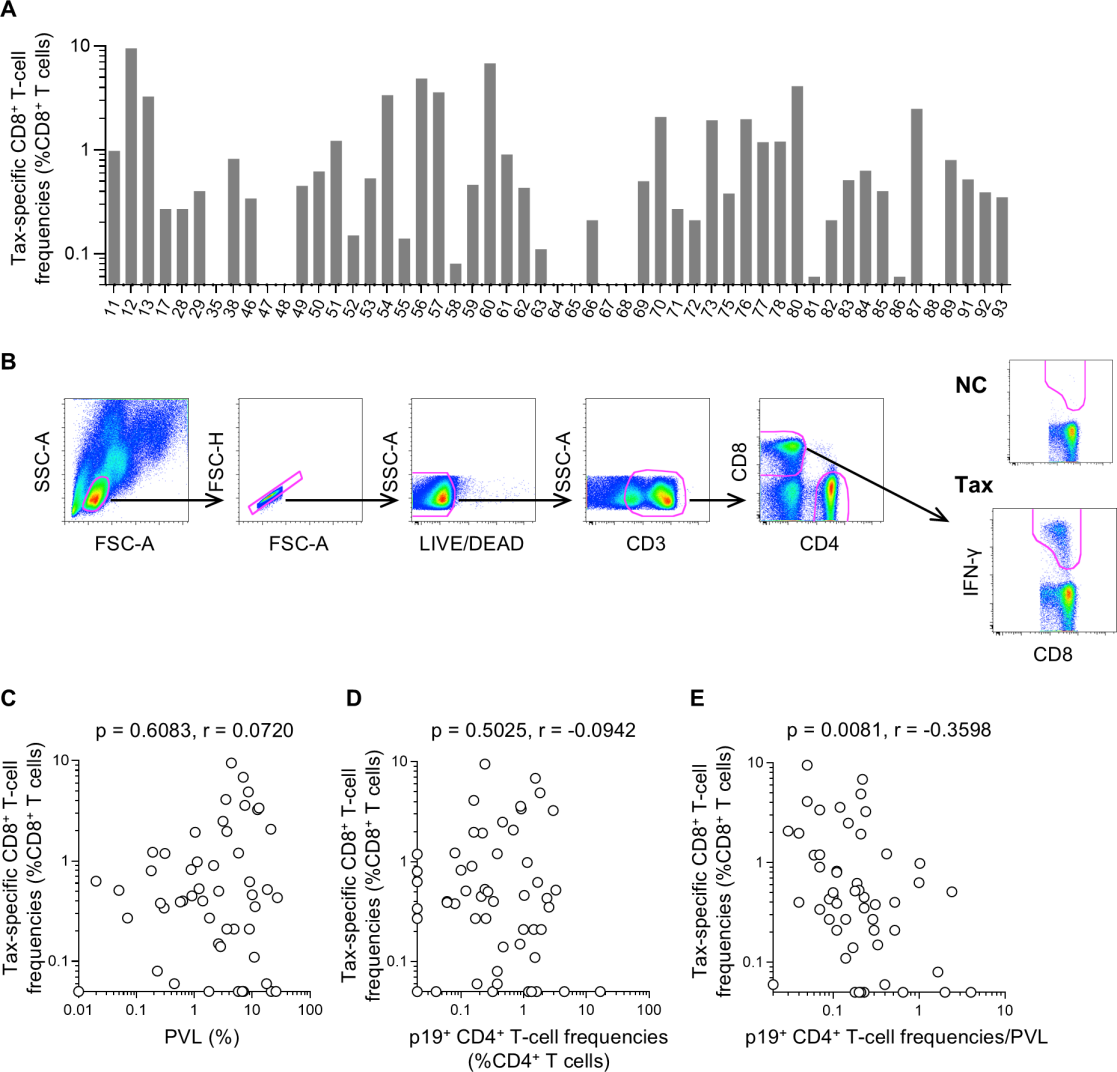
Tax-specific CD8^+^ T-cell responses in asymptomatic HTLV-1 carriers. (**A**) Tax-specific CD8^+^ T-cell frequencies in carriers (*n* = 53). (**B**) A representative gating schema for the detection of specific IFN-γ induction after Tax-specific stimulation in flow cytometric analysis. Data on PBMCs of sample #12 are shown. The CD8-IFN-γ dot plot without stimulation is also shown (NC). (**C**) Correlation between PVLs and Tax-specific CD8^+^ T-cell frequencies. (**D**) Correlation between p19-expressing CD4^+^ T-cell frequencies and Tax-specific CD8^+^ T-cell frequencies. (**E**) Correlation between ratios of p19-expressing CD4^+^ T-cell frequencies to PVLs and Tax-specific CD8^+^ T-cell frequencies.

Finally, virus-specific CD8^+^ T-cell responses were examined. Tax is the most immunogenic target antigen for CD8^+^ T cells among HTLV-1 viral proteins, and the contribution of Tax-specific CD8^+^ T-cell responses to HTLV-1 control has been indicated (21–25). Thus, Tax-specific CD8^+^ T-cell responses were investigated. Using freshly prepared PBMCs from asymptomatic HTLV-1 carriers (*n* = 53), Tax-specific CD8^+^ T-cell responses were analyzed. Tax-specific CD8^+^ T-cell responses were detectable in 45 of 53 carriers (Fig. 5A and B). No correlation was observed between PVLs and Tax-specific CD8^+^ T-cell frequencies (*P* = 0.6083, *r* = 0.0720 by Spearman’s test) (Fig. 5C). Similarly, no correlation was observed between p19-expressing CD4^+^ T-cell frequencies after overnight cell culture and Tax-specific CD8^+^ T-cell frequencies (*P* = 0.5025, *r* = -0.0942 by Spearman’s test) (Fig. 5D). Interestingly, a majority of carriers with high (>1%) Tax-specific CD8^+^ T-cell frequencies showed high (> 1%) PVLs but low (<1%) p19-expressing CD4^+^ T-cell frequencies. Indeed, Tax-specific CD8^+^ T-cell frequencies were inversely correlated with the ratio of p19-expressing CD4^+^ T-cell frequencies to PVLs (*P* = 0.0081, *r* = −0.3598 by Spearman’s test) (Fig. 5E).

## DISCUSSION

In the acute phase of HTLV-1 infection, viral expression and replication occur. Viral protein expression results in the induction of host immune responses, and some of these immune responses contribute to the control of the spread of infected cells, implying virus-host immune interaction. It is speculated that these virus-host immune interactions are involved in determining the scale of infection or PVL in the acute phase of HTLV-1 infection.

In the asymptomatic chronic phase, the virus-host immune interaction may not be strong because viral protein expression is poor in latent infection. However, marginal viral protein expression has been suggested in the latent phase (10, 27). Therefore, virus-host immune interaction may persist, although not strong, while the virus-host immune relationship in the asymptomatic chronic phase has not been fully determined. To address this issue, the present study performed virological and immunological analyses using PBMCs derived from asymptomatic HTLV-1 carriers.

PVL has been considered a predictive marker for HTLV-1-associated disease progression (28–31). Cumulative studies have examined the relationship between PVL and host immune responses (32–35), but it has not yet been clearly understood. In the present cross-sectional study, we analyzed PVL, HTLV-1 antigen expression after *ex vivo* PBMC culture, binding antibodies, NAb responses, and T-cell responses in asymptomatic HTLV-1 carriers. This study, assessing the relationship among all of these, would be important in providing data to contribute to our understanding of virus-host immune interaction in asymptomatic HTLV-1 infection.

In the present study, asymptomatic HTLV-1 carriers with higher PVLs had higher levels of anti-HTLV-1 binding antibodies. Higher anti-HTLV-1 antibody levels were associated with higher anti-HTLV-1 NAb responses. The association of PVL with anti-HTLV-1 NAb activity has not been clearly shown (36), but our analysis revealed a significant positive correlation between PVL and anti-HTLV-1 NAb activity in asymptomatic HTLV-1 carriers. These results reflect the dependency of the magnitudes of induced anti-HTLV-1 NAb responses on antigen expression levels, which are associated with PVLs, suggesting that the contribution of anti-HTLV-1 NAb responses to the control of HTLV-1 replication is not large.

HTLV-1 antigen expression was confirmed mostly after overnight PBMC culture. Frequencies of p19-expressing CD4^+^ T cells were positively correlated with PVLs (*r* = 0.8486). This indicates p19 expression in a majority of infected cells after *ex vivo* culture, implying that a majority of latently infected cells whose viral antigen expression is suppressed *in vivo* have the potential to express viral antigens *ex vivo*. Non-specific IFN-γ induction in CD8^+^ T cells was confirmed in all samples after overnight PBMC culture. Frequencies of IFN-γ^+^ CD8^+^ T cells after overnight culture were positively correlated not only with PVLs but also with p19-expressing CD4^+^ T-cell frequencies. These results suggest that IFN-γ was induced in CD8^+^ T cells by stimulation with HTLV-1 antigens expressed during overnight culture.

In the present study, Tax-specific CD8^+^ T-cell responses were detected in 45 of 53 asymptomatic HTLV-1 carriers. Previous studies indicated that HTLV-1 antigen-specific CD8^+^ T-cell responses, in particular, Tax-specific CD8^+^ T-cell responses, play a central role in the control of HTLV-1 replication (37–39). Association of Tax-specific CD8^+^ T-cell responses with PVLs has been indicated in some reports (21, 22, 24, 32). However, the relationship between PVLs and Tax-specific CD8^+^ T-cell responses may be elusive if Tax-specific CD8^+^ T-cell responses are enhanced by Tax expression but suppress Tax expression. Indeed, in contrast to anti-HTLV-1 NAb activity, Tax-specific CD8^+^ T-cell frequencies did not significantly correlate with PVLs in the present study. No significant association was observed between Tax-specific CD8^+^ T-cell frequencies and p19-expressing CD4^+^ T-cell frequencies. However, a majority of carriers with high (>1%) Tax-specific CD8^+^ T-cell frequencies showed high (>1%) PVLs (Fig. 5C) but low (<1%) p19-expressing CD4^+^ T-cell frequencies (Fig. 5D), although p19-expressing CD4^+^ T-cell frequencies were positively correlated with PVLs (Fig. 3D). These results suggest the suppression of p19 expression in infected cells by Tax-specific CD8^+^ T-cell responses. Further analysis found a significant inverse correlation between Tax-specific CD8^+^ T-cell frequencies and the ratio of p19-expressing CD4^+^ T-cell frequencies to PVLs (Fig. 5E). The ratio of p19-expressing cell frequencies to PVLs reflects the frequency of cells producing viral antigens in infected cells. This provides evidence indicating that Tax-specific CD8^+^ T-cell responses are responsible for the suppression of HTLV-1 p19 expression in *ex vivo* culture. It is inferred that this inhibitory effect of Tax-specific CD8^+^ T-cell responses on HTLV-1 antigen expression can be exerted in virus-host immune interaction in asymptomatic HTLV-1 carriers.

This cross-sectional study was not able to assess changes in virus-host immune interaction. Elucidation of predictive markers for disease progression by longitudinal analyses remains to be the next issues.

In summary, PVL, viral antigen expression in *ex vivo* culture, antibody responses, and T-cell responses were investigated in asymptomatic HTLV-1-infected carriers in this study. Anti-HTLV-1 NAb responses were positively correlated with PVLs. Tax-specific CD8^+^ T-cell frequencies were not associated with PVLs but inversely correlated with the ratios of p19-expressing CD4^+^ T-cell frequencies to PVLs, implicating Tax-specific CD8^+^ T cells in the control of HTLV-1 replication. These results provide insights into the elucidation of the virus-host immune interaction in latent HTLV-1 infection.

## MATERIALS AND METHODS

### Study participants

This study enrolled HTLV-1-infected asymptomatic carriers (*n* = 77), who sought medical care at the Institute of Medical Science Health Facility, University of Tokyo, Japan, from 2018 to 2021. These participants had been diagnosed as HTLV-1 positive through screening by chemiluminescent immunoassay or chemiluminescent enzyme immunoassay (Lumipulse Fujirebio, ARCHITECT HTLV Abbott), followed by line immunoassay (INNO-LIA HTLV I/II, Fujirebio) for the confirmation of positive results by excluding false positives, and were enrolled in the program of Joint Study on Predisposing Factors of ATL Development (JSPFAD). All enrolled donors were over 18 years old and provided written consent according to the Ethical Guidelines for Medical and Biological Research Involving Human Subjects established by the Ministry of Education, Culture, Sports, Science and Technology, the Ministry of Health, Labour and Welfare, and the Ministry of Economy, Trade and Industry in Japan (https://www.mhlw.go.jp/content/001077424.pdf). Clinical information on age, sex, and PVLs was obtained via JSPFAD from the participants’ medical records. PBMCs were prepared by density gradient centrifugation using a Ficoll-Paque (Cytiva). Plasma samples were heat inactivated at 56°C for 30 min.

### Quantification of proviral loads

PVLs in PBMCs were quantified by real-time TaqMan quantitative polymerase chain reaction (PCR) using the ABI Prism 7000 sequence detection system (Applied Biosystems) as previously described (16, 40). Briefly, genomic DNA (50 ng) extracted from PBMCs using a Qiagen Blood Kit (Qiagen) was subjected to real-time PCR using primers targeting the HTLV-1 pX region and the human RNase P gene (Applied Biosystem) for the internal control to calculate input cell numbers. DNAs extracted from TL-Om1 (an ATL-derived cell line) (41) and normal healthy PBMCs were used as positive and negative controls, respectively. The HTLV-1 proviral loads were expressed as the copy number per 100 PBMCs, assuming each infected cell contains one integrated HTLV-1 provirus copy. Data deviations were corrected in each experiment by adjusting the positive control (TL-Om1) to 100% and proportionally recalibrating sample data.

### Analysis of binding antibodies

Anti-HTLV-1 antibodies were assessed by line immunoassay according to the manufacturer’s instructions. In brief, 10 µL of heat-inactivated plasma was added to 1 mL of diluent and incubated overnight at room temperature with HTLV-1 antigen-coated strips, followed by subsequent incubation with a conjugate and substrate solution. The scores for relative levels of antibodies specific for individual HTLV-1 antigens (Gag [p19 and p24] and Env [gp46 and gp21]) were determined based on the band (line) intensity according to the manufacturer’s instructions and shown as 0 (negative), 1+, 2+, 3+, or 4+ (highest).

### Analysis of anti-HTLV-1 neutralizing antibody activity

Anti-HTLV-1 NAb activity was assessed by measuring syncytia inhibition (42). HTLV-1-producing cells (ATL040 cells provided by Y. Tanaka [43]) were pre-incubated with diluted heat-inactivated plasma at 37°C for 30 min and cocultured with Jurkat cells. One day later, the number of syncytia was counted in nine microscopic fields per well. Syncytia-forming activity was calculated as the ratio of the syncytia count to that of the control culture without plasma, and neutralization activity was determined as the inhibitory ratio of syncytia-forming activity.

### Analysis of p19 expression and interferon-γ induction in T cells

Freshly prepared PBMCs were separated into three groups. The first group was immediately (without culture) subjected to analyses of HTLV-1 Gag matrix p19 antigen expression, IFN-γ induction, and Tax-specific CD8^+^ T-cell responses. The second group was cultured overnight and subjected to analyses of p19 expression and IFN-γ induction. The remaining third was frozen for stock. For analyses of p19 expression and IFN-γ induction in PBMCs, intracellular staining was performed using a Cytofix Cytoperm kit (BD Biosciences) with a LIVE/DEAD Fixable Aqua Dead Cell Stain Kit (Invitrogen), anti-CD3 APC-Cy7 (SP34-2; BD Biosciences), anti-CD4 FITC (M-T477; BD Biosciences), anti-CD8 PerCP (SK1; BD Biosciences), and anti-p19 (TP-7; Abcam) conjugated with AlexaFluor 647 (AF647) using AF647 Antibody Labeling kit (Molecular Probes) or anti-IFN-γ PE (4S. B3; BioLegend). Stained cells were analyzed by BD FACS Canto II with FACS Diva version 8.0.1 (BD Biosciences) and FlowJo version 9.2 (FlowJo). For individual FACS analyses, 2.5–5.9 × 10^5^ cells were incorporated.

### Analysis of Tax-specific T-cell responses

HTLV-1 Tax-specific CD8^+^ T-cell frequencies were measured by flow cytometric analysis of IFN-γ induction after specific stimulation. As described above, freshly prepared PBMCs were pulsed with peptide pools (at a final concentration of 2 µM for each peptide) using panels of overlapping peptides spanning HTLV-1 Tax amino acid sequences in the presence of GolgiPlug (Brefeldin A; BD Biosciences) and 1 µg/mL co-stimulatory anti-CD28 antibody (CD28.2; BD Biosciences) for 6 hours. Intracellular IFN-γ staining was performed as described above. Stained cells were analyzed by BD FACS Canto II with FACS Diva version 8.0.1 and FlowJo version 9.2. For individual FACS analyses, 1.8–6.1 × 10^5^ cells were incorporated. Specific CD8^+^ T-cell frequencies were calculated by subtracting nonspecific IFN-γ^+^ CD8^+^ T-cell frequencies from those after peptide-specific stimulation. Specific CD8^+^ T-cell frequencies less than 0.05% of CD8^+^ T cells were considered negative.

### Statistical analysis

Statistical analyses were performed using Prism software (GraphPad Software, Inc.) with significance set at *P* values of <0.05. Comparisons were performed by Mann-Whitney *U* test. Correlation analyses were performed by Spearman’s test.

## Data Availability

All data supporting the conclusion in this study are included in the main text and figures, and additional information is available from the corresponding author upon request.
